# Early Pain and Sensory Dysfunction in Drug‐Naïve Parkinson's Disease: Evidence From Quantitative Sensory Testing

**DOI:** 10.1002/ejp.70403

**Published:** 2026-09-23

**Authors:** Maria Skallerup Andersen, Lorenz Martin Oppel, Boris Modrau, Kimmo Jensen, Gertrud Dam‐Dalgeir, Parisa Gazerani

**Affiliations:** ^1^ Department of Neurology Aalborg University Hospital Aalborg Denmark; ^2^ Department of Clinical Medicine Aalborg University Aalborg Denmark; ^3^ FORSA, Forskningens Hus Aalborg Denmark; ^4^ Department of Life Sciences and Health, Faculty of Health Sciences Oslo Metropolitan University Oslo Norway; ^5^ Department of Health Science and Technology, Faculty of Medicine Aalborg University Aalborg Denmark

**Keywords:** dynamic mechanical allodynia, mechanical hyperalgesia, nociceptive processing, people with drug‐naïve Parkinson's disease, quantitative sensory testing, sensory disturbances

## Abstract

**Background:**

Pain and sensory disturbances are common non‐motor symptoms in Parkinson's disease (PD) and can occur early in the disease course, even before starting dopaminergic treatment. However, somatosensory abnormalities in drug‐naïve PD remain insufficiently characterized, particularly in the absence of dopaminergic medication effects.

**Methods:**

In this cross‐sectional study, 35 people with drug‐naïve PD (PwP) and 35 individually age‐ and sex‐matched healthy controls underwent quantitative sensory testing (QST). Testing included brush stimulation to assess dynamic mechanical allodynia (DMA), pinprick stimulation to assess mechanical pain sensitivity—both rated using a numeric rating scale (NRS)—and pressure pain threshold (PPT) measurements. PwP were stratified by daily pain status. Data were analysed using linear mixed‐effects models (LMEMs) that accounted for matching and repeated measures.

**Results:**

Pinprick stimulation revealed statistically significant mechanical hyperalgesia across all tested body regions in PwP, regardless of pain status, compared with healthy controls. PPT testing demonstrated significantly lower thresholds in PwP across most body regions, indicating early pressure hyperalgesia. In contrast, brush testing did not reveal significant group differences, and only a small proportion of PwP showed signs of DMA. No significant differences were observed between PwP with and without pain, nor between the disease‐onset side and the contralateral side.

**Conclusions:**

Drug‐naïve PD is characterized by early nociceptive sensory abnormalities, with consistent evidence of mechanical hyperalgesia even in patients without chronic pain. The absence of DMA suggests that nociceptive hypersensitivity may precede alterations in non‐nociceptive processing. Nociceptive QST measures may therefore represent indicators of altered sensory processing in early‐stage PwP.

**Significance Statement:**

This study adds to existing evidence that sensory disturbances, including pain, may be present already in the early, drug‐naïve stages of Parkinson's disease. Clinically, these findings highlight the importance of recognizing pain and sensory abnormalities as potential early non‐motor features of PD, including before dopaminergic treatment is initiated.

**Abbreviations**
DMA: dynamic mechanical allodynia
EMMs: estimated marginal means
LMEM: linear mixed‐effects model
MDI‐D: Danish version of the Major Depressive Inventory
MoCA‐D: Danish version of the Montreal Cognitive Assessment
NRS: numeric rating scale
PD: Parkinson's disease
PD‐PCS: Parkinson's Disease Pain Classification System
PPT: pressure pain threshold
PwP: people with drug‐naïve Parkinson's disease
PwPD: people with Parkinson's disease
QST: quantitative sensory testing
UPDRS: Unified Parkinson's Disease Rating Scale

## 1 Introduction

Non‐motor symptoms in people with Parkinson's disease (PwPD), including sensory disturbances and altered pain perception, are increasingly recognized as contributors to disease burden (Silverdale et al. 2018). Pain affects approximately 55%–72% of people with early PD (Baig et al. 2015; Mylius et al. 2016; Boura et al. 2017) and 20%–59% experience pain before visible motor symptoms (Pont‐Sunyer et al. 2015; Defazio et al. 2017). Pain presentations in PD are complex, encompassing musculoskeletal, dystonic, radicular‐peripheral neuropathic, central neuropathic pain and akathisia (Ford 2010). More recently, the Parkinson's Disease Pain Classification System (PD‐PCS) differentiates PD‐related from non‐PD‐related pain and classifies PD‐related pain (Mylius et al. 2021; Tinazzi et al. 2025). Basal ganglia dysfunction is thought to contribute to sensory impairments and pain (Brefel‐Courbon et al. 2005; Wasner and Deuschl 2012). Altered central nociceptive processing has also been reported in early PD (Tseng and Lin 2017). However, mechanisms underlying pain in early PD, including de novo and drug‐naïve stages, remain poorly understood.

Quantitative sensory testing (QST) is a standardized method for assessing sensory function and abnormalities in pain processing, including peripheral and central alterations that may precede motor symptoms. Studying drug‐naïve patients enables investigation of sensory and pain‐related changes without the confounding effects of dopaminergic medication, which is important for understanding the early pathophysiology of nociceptive and potentially chronic pain in PD.

QST studies in late‐stage PD generally report increased sensitivity to sensory and painful stimuli compared with healthy controls (Mylius et al. 2009; Zambito Marsala et al. 2011). However, research in early‐stage PD is limited, and findings are inconsistent. Variability may reflect methodological challenges in assessing sensory abnormalities in the OFF‐state, including differences in medication washout and half‐lives, as well as small sample sizes (maximum 21 PwPD and 23 healthy controls) (Mylius et al. 2011; Petschow et al. 2016; Forkmann et al. 2017). Five studies investigated early‐stage PwPD in the OFF‐state (Mylius et al. 2011, 2016; Petschow et al. 2016; Boura et al. 2017; Forkmann et al. 2017) and one in the ON‐state (Andersen et al. 2015).

Findings remain heterogeneous; one study reported increased sensitivity to brushstroke and pinprick, reduced pressure pain threshold (PPT) before and after the cold pressure threshold test in conditioned pain modulation and reduced cold pressure threshold tolerance time (Andersen et al. 2015). Other studies found no differences in electrical pain, heat pain or warmth perception thresholds (Mylius et al. 2011, 2016; Petschow et al. 2016; Boura et al. 2017; Forkmann et al. 2017). Only one QST study in people with drug‐naïve PD (PwP) reported no differences compared with controls or between PwP with and without pain (Fründt et al. 2019). Overall, evidence in drug‐naïve and early PD remains limited and inconsistent, requiring further investigation.

This study investigated sensory responses and pain perception in PwP using QST. Comparisons between PwP and healthy controls and between PwP with and without pain aimed to characterize sensory dysfunction and nociceptive abnormalities and explore potential peripheral and central sensory alterations. The findings may improve understanding of pain pathophysiology and early non‐motor symptoms in PD and inform the development of targeted interventions for pain management.

## 2 Materials and Methods

### 2.1 Study Design

This cross‐sectional observational study was approved by the Ethical Committee of the North Denmark Region (N‐20170025) and conducted in accordance with the Helsinki Declaration. The project was reported to The Danish Data Protection Agency via the North Denmark Region umbrella agreement. Written informed consent was obtained from PwP and healthy controls before participation. PwP were assessed at baseline before the initiation of dopaminergic treatment and re‐examined after 3 and 12 months of medication. The present sub‐study focuses on baseline sensory abnormalities in PwP and was conducted between June 2017 and July 2019 as an integral component of the extensive project titled ‘The Aalborg PD Pain Project’ (June 2017–July 2020), a prospective longitudinal observational cohort study. No a priori sample size calculation was performed. The study size was determined by the number of eligible participants recruited during the available recruitment period from June 2017 to July 2019.

### 2.2 Study Participants

To recruit PwP, all neurological referrals to the Department of Neurology at Aalborg University Hospital, as well as referrals from neurologists in private practice in Jutland, Denmark, were reviewed. All participants with suspected PD underwent a neurological examination and clinical evaluation by a neurologist specialized in movement disorders. The primary investigator participated in the screening and recruitment procedures to ensure eligibility for study participation. Participants were diagnosed with PD according to both the UK Parkinson's Disease Society Brain Bank Clinical Diagnostic Criteria (Hughes et al. 1992) and the Movement Disorder Society Clinical Diagnostic Criteria for Parkinson's disease (MDS‐PD Criteria; Postuma et al. 2015). PwP underwent longitudinal follow‐up for 12 months as part of The Aalborg PD Pain Project, and individuals whose diagnosis was later reconsidered were excluded from the present study.

After consultations with the neurologist, PwP received an invitation to participate if they were ≥ 18 years old, newly diagnosed (≤ 1 week), not previously treated with PD medication and not taking other medications except cholesterol‐lowering drugs, heart medication (e.g., antihypertensives and anticoagulants), metabolic medications, diuretics and/or vitamins. Participants had to demonstrate normal cognitive function and activities of daily living, having no diagnosis of diabetes mellitus or other central or peripheral nervous system disorders, no history of psychiatric disorders and no history of drug or alcohol addiction. Following neurological screening, written informed consent was obtained. All study assessments were completed within 7 days of PD diagnosis confirmation, and dopaminergic treatment was initiated only after completion of the study assessments. Participants then attended a single 2‐h session at the outpatient clinic of the Department of Neurology at Aalborg University Hospital, Denmark.

To meet the final inclusion criteria, PwP underwent mental and physical assessments performed by the primary investigator. Cognitive criteria included achieving ≥ 28 on the Danish version of the Montreal Cognitive Assessment (MoCA‐D) (Nasreddine et al. 2005; Robotham et al. 2020). Although a score of 27 was accepted for PwP with ≤ 12 years of education according to standard MoCA education adjustment guidelines, a cut‐off of 28 was applied in the present study. In addition, the absence of depression was required, assessed using the Danish version of the Major Depressive Inventory (MDI‐D) (Bech et al. 2001, 2015), according to the ICD‐10 algorithm for depression diagnosis. Physical assessments included the Unified Parkinson's Disease Rating Scale (UPDRS) (Martínez‐Martín et al. 1994), including Part I (Mentation, Behaviour and Mood) and Part III (Motor Examination). Disease severity was assessed by the Hoehn and Yahr staging scale (Hoehn and Yahr 1967) and activities of daily living were assessed using the Schwab and England Activities of Daily Living Scale (Martínez‐Martín et al. 1994). For the final inclusion criteria, participants were required to have Hoehn and Yahr Stage I–III and a Schwab and England score ≥ 70%. These requirements ensured that PwP were able to understand the tasks and evaluate the perception of painful stimulation. In addition, PwP were excluded if they had taken pain medication or consumed alcohol within 24 h before the examination.

The PwP were divided into two groups based on the presence or absence of self‐reported daily pain, assessed through a structured clinical interview. Participants reporting daily pain were asked about the location and duration of the pain and whether it could be attributed to a previous injury or another non‐PD‐related cause. Pain attributed to a previous injury or another non‐PD‐related cause was excluded. Group 1 included patients with chronic pain and sensory disturbances considered to be related to PD, defined as daily pain lasting more than 3 months but no longer than 5 years. Group 2 consisted of patients who did not experience daily pain.

**FIGURE 1:**
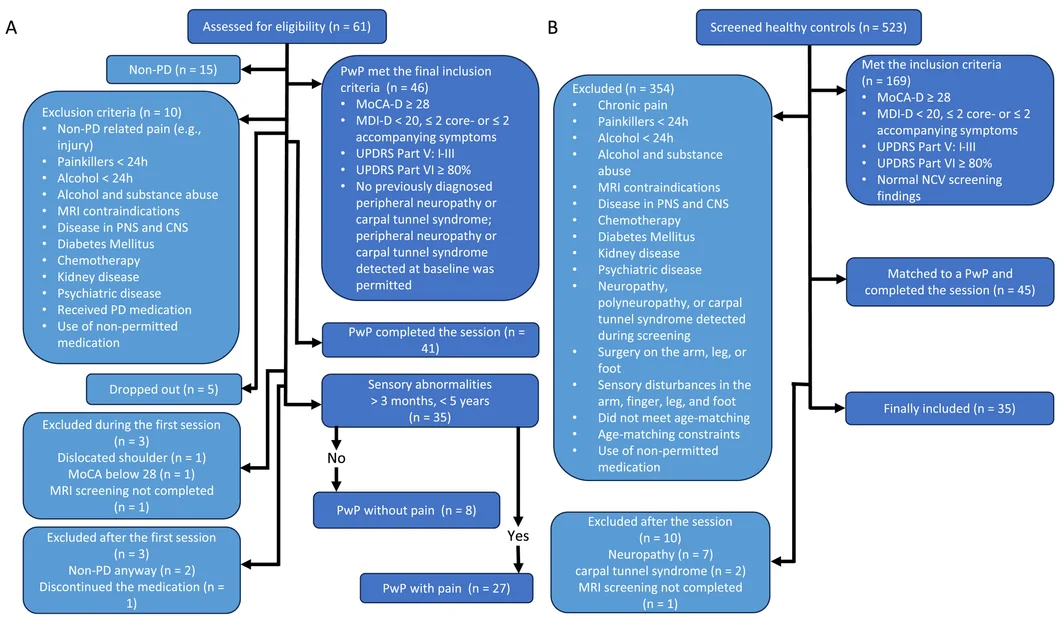
A flowchart of participant recruitment, screening, exclusion and inclusion. (A) People with drug‐naïve Parkinson's disease and (B) healthy controls. NCV investigation and MRI of the cerebrum were performed as part of the clinical screening procedure within the Aalborg PD Pain Project and are included in the flowchart only to illustrate the screening process and reasons for exclusion. NCV and MRI data were not analysed as part of the present QST study. CNS, central nervous system; MDI‐D, Danish version of the Major Depressive Inventory; MoCA‐D, Danish version of the Montreal Cognitive Assessment; MRI, magnetic resonance imaging; NCV, nerve conduction velocity; PD, Parkinson's disease; PNS, peripheral nervous system; PwP, people with drug‐naïve Parkinson's disease; UPDRS, Unified Parkinson's Disease Rating Scale.

To evaluate changes in MoCA, MDI and QST parameters within the PwP groups, healthy controls were recruited through advertisements. Each PwP was matched with a healthy control by age (±2 years) and sex (see Figure 1). The inclusion and exclusion criteria for healthy controls were identical to those for PwP, except for the UPDRS Parts I and III, and Hoehn and Yahr Staging Scale and activities of daily living. Healthy controls were additionally required not to have experienced pain for more than 3 months in the same areas, nor to have any history of injury or surgery in the tested regions.

### 2.3 Evaluation of Pain Perception

All QST assessments were conducted by the primary investigator, who was trained in standardized QST procedures to minimize inter‐rater variability. Due to the study design and recruitment procedures, this investigator was not blinded to participant group.

Testing was performed in a quiet room with the lights on and the window closed, and the room temperature was maintained between 20°C and 23°C. Before testing, participants completed a brief practice trial with a few brushstrokes, pinprick stimulators and algometer presses to familiarize themselves with the procedure. None of the participants had previous experience with sensory testing.

During the examination, participants were assessed for tactile and pain sensitivity as well as pain thresholds. To maintain full concentration, they were instructed to keep their eyes closed throughout the session. Testing was performed bilaterally in a fixed, region‐by‐region order, with the right side tested first, followed by the left within each body region (i.e., right neck, left neck; right arm, left arm; right leg, left leg; right foot, left foot). This order was applied consistently across all participants, irrespective of the disease‐onset side.

Body sites were selected based on clinical relevance to PD‐related pain and prior findings on PwPD (Zambito Marsala et al. 2011; Petschow et al. 2016). First, pain stimuli were applied to the lower arms (centrally between the elbow and wrist), while participants sat with their forearms resting on a table in a supinated position. Second, stimuli were applied to the neck, with participants seated comfortably and their arms resting on their thighs. Finally, pain stimuli were applied to the foot, while participants placed their feet on a stool to allow a relaxed position.

Tactile stimuli were administered first to the lower arms (between the elbow and wrist), followed by the lower legs (between the knee and ankle) and finally to the foot (between the ankle and toes).

After each mechanical test, participants rated their discomfort or pain on a numeric rating scale (NRS) from 0 (‘no pain’) to 10 (‘worst possible pain’), a tool chosen for its reliability, ease of use and clinical validity.

### 2.4 Quantitative Sensory Testing

QST is a standardized psychophysical approach for assessing somatosensory function using a range of validated sensory modalities. In this study, three selected QST measures (dynamic mechanical allodynia [DMA], pinprick stimulation and PPT) were applied, rather than the complete standardized protocol.

#### 2.4.1 Response of Dynamic Mechanical Allodynia to Light Manual Brush

The brush test was used to evaluate the stimulus–response function to non‐painful stimuli and skin sensitivity, as well as to identify sensory abnormalities such as allodynia.

A standardized handheld brush (SENSELab Brush‐05; Somedic, Hörby, Sweden) was applied across the skin in a single stroke, repeated three times per area from proximal to distal. Each stroke had an inter‐stimulus interval of 5 s, with a speed of 1–2 cm/s and an approximate angle of 45°. Stroke lengths were ~20 cm on the lower arms, ~30 cm on the legs and ~10 cm on the foot. Brush stimulation was considered non‐nociceptive, and pain ratings in response to brush stimulation were interpreted as allodynia.

Participants rated pain intensity after each stroke on the NRS, and DMA was operationally defined as an NRS score ≥ 1.

#### 2.4.2 Response of Mechanical Pain Sensitivity to Pinprick Stimulation

Pinprick stimulators were used to evaluate stimulus–response functions for painful stimuli and detect abnormalities such as hyperalgesia. The validated set (Pinprick Stimulator Set; MRC System GmbH, Heidelberg, Germany) consists of seven weighted flat‐tipped needles (8, 16, 32, 64, 128, 256 and 512 mN) with a contact area of 0.2 mm^2^ (Rolke et al. 2006).

Stimuli were applied individually in random order within a circle with a diameter of 3 cm to avoid repeated stimulation of the same spot. Each stimulator was applied three times with an inter‐stimulus interval of 2–4 s at an angle of ~90° (lower arm, neck, foot). Participants rated pain intensity for each stimulus on the NRS. For each stimulus intensity, the mean NRS score across the three repetitions was used for analysis.

#### 2.4.3 Pressure Pain Threshold

Pressure algometry was used to assess sensitivity to mechanical pressure stimuli and to evaluate PPT. PPT was assessed using a handheld pressure algometer (Somedic, Hörby, Sweden) with a gun‐shaped holder and a 1 cm^2^ rubber‐covered probe. PPT was determined using three series of ascending stimulus intensities, increasing at a rate of 30 kPa/s. Pressure values were displayed digitally.

Participants held a stop button and were instructed to press it when pressure first transitioned to pain. The corresponding value was recorded as the PPT (kPa). PPT measurements were applied at ~90° to each body area (lower arm, neck, foot) with 60‐s inter‐stimulus intervals. The mean PPT value across the three measurements was used for analysis.

### 2.5 Statistical Analysis

Data were recorded and managed using REDCap (version 13.1.37, North Denmark Region). Statistical analyses and graphing were performed in R (version 4.3.3; R Foundation for Statistical Computing, Vienna, Austria) using the nlme package for linear mixed‐effects models (LMEMs) and the emmeans package for estimated marginal means (EMMs).

Baseline participant characteristics were compared between groups using Fisher's exact test for categorical variables and unpaired *t*‐tests for continuous variables.

Given the limited number of pathological brush test scores (NRS ≥ 1), differences in brush‐evoked pain between groups were assessed using Fisher's exact test.

LMEMs were used to analyse pinprick and PPT data for each anatomical location separately. For the pinprick analyses, participant group and pressure level were included as fixed effects, whereas participant group was included as the sole fixed effect in the PPT analyses. Separate models assessing side‐related differences within the PD groups replaced participant group with side (disease‐onset side vs. contralateral side) as the fixed effect. Random intercepts for matched pairs and participants nested within matched pairs were included to account for the matched study design and repeated measurements. Post hoc pairwise comparisons were adjusted using Tukey's method. Statistical significance was defined as *p* < 0.05. Detailed statistical methods are provided in Methods S1.

## 3 Results

PwP are presented as a combined group in Tables 1 and 2 and stratified according to the presence of self‐reported PD‐related daily pain (PwP with pain vs. without pain) in Tables 3 and 4.

**TABLE 1:** Demographic and clinical characteristics of PwP and healthy controls.

Characteristics	Healthy controls (*n* = 35)	PwP with and without pain (*n* = 35)
Age, years (mean ± SD; min–max)	64.7 ± 10.3 (38–81)	64.8 ± 10.3 (38–82)
Sex, *n* (%)		
Female	17 (48.6)	18 (51.4)
Male	18 (51.4)	17 (48.6)
Pain, *n* (%)		
No	35 (100.0)	8 (22.9)
Yes	—	27 (77.1)
Disease‐onset side, *n* (%)		
Right	—	18 (51.4)
Left	—	17 (48.6)
Time since PD diagnosis, days	—	≤ 7
MoCA score (mean ± SD)	29.4 ± 0.8	29.1 ± 0.8
MoCA score, *n* (%)		
28	6 (17.1)	10 (28.6)
29	8 (22.9)	12 (34.3)
30	21 (60.0)	13 (37.1)
MDI rating scale score (mean ± SD)	2.0 ± 3.8 (0–17)	5.0 ± 5.6 (0–19)
MDI ICD‐10, *n* (%)		
0	35 (100.0)	35 (100.0)
MDI ICD‐10 algorithm core symptoms, *n* (%)		
0	35 (100)	32 (91.4)
1	0 (0.0)	3 (8.6)
MDI ICD‐10 algorithm accompanying symptoms, *n* (%)		
0	35 (100)	31 (88.6)
1	0 (0.0)	2 (5.7)
2	0 (0.0)	2 (5.7)
UPDRS Part I (mean ± SD)	—	1.09 ± 1.12
UPDRS Part III (mean ± SD)	—	16.80 ± 4.90
Hoehn and Yahr staging (mean ± SD)	—	1.51 ± 0.56
Hoehn and Yahr stage, *n* (%)		
1	—	18 (51.4)
2	—	16 (45.7)
3	—	1 (2.9)
Schwab and England Activities of Daily Living Scale (mean ± SD)	100 ± 0.00	87.71 ± 4.90

*Note:* PwP were stratified into two groups based on self‐reported PD‐related daily pain lasting more than 3 months (hereafter referred to as ‘PwP with pain’ and ‘PwP without pain’). Disease‐onset side applies only to PwP. Parkinson's disease‐specific measures, including UPDRS scores, Hoehn and Yahr stage, disease‐onset side and Activities of Daily Living, apply only to PwP and are therefore not reported for healthy controls.

Abbreviations: MDI‐D, Danish version of the Major Depressive Inventory; MoCA‐D, Danish version of the Montreal Cognitive Assessment; PD, Parkinson's disease; PwP, people with drug‐naïve Parkinson's disease; SD, standard deviation; UPDRS, Unified Parkinson's Disease Rating Scale.

**TABLE 2:** Responses to light brush stimulation in PwP with and without pain and healthy controls.

Body region	PwP NRS ≥ 1 (*n*/*N*, %)	Healthy control NRS ≥ 1 (*n*/*N*, %)	Absolute difference (%)	Comparison *p*‐value
Right arm	3/35 (8.6)	0/35 (0.0)	8.6	0.24
Left arm	5/35 (14.3)	0/35 (0.0)	14.3	0.05
Right leg	3/35 (8.6)	0/35 (0.0)	8.6	0.24
Left leg	5/35 (14.3)	0/35 (0.0)	14.3	0.05
Right foot	5/35 (14.3)	1/35 (2.9)	11.4	0.20
Left foot	4/35 (11.4)	0/35 (0.0)	11.4	0.11

*Note:* Values represent the number and percentage of participants reporting brush‐evoked pain or discomfort, defined as a numeric rating scale score ≥ 1 (NRS ≥ 1). Absolute percentage differences between groups are presented together with corresponding *p*‐values from Fisher's exact test comparing the combined PwP group and healthy controls.

Abbreviations: NRS, numeric rating scale; PwP, people with Parkinson's disease.

**TABLE 3:** Estimated marginal mean (EMM) differences in static mechanical pain sensitivity (pinprick testing) across participant groups and body regions.

Body region	PwP with pain (*n* = 27) vs. healthy controls (*n* = 27)	PwP without pain (*n* = 8) vs. healthy controls (*n* = 8)	PwP with pain (*n* = 27) vs. PwP without pain (*n* = 8)
Right neck	−1.35 (−1.87, −0.83), < 0.0001^a^	−1.51 (−2.36, −0.67), 0.0003^a^	0.16 (−0.73, 1.06), 0.89
Left neck	−1.30 (−1.91, −0.70), < 0.0001^a^	−1.88 (−2.84, −0.92), 0.0001^a^	0.58 (−0.42, 1.58), 0.34
Right arm	−0.98 (−1.50, −0.46), 0.0002^a^	−1.16 (−2.01, −0.30), 0.01^a^	0.17 (−0.73, 1.08), 0.88
Left arm	−1.27 (−1.83, −0.70), < 0.0001^a^	−1.31 (−2.23, −0.40), 0.004^a^	0.05 (−0.93, 1.02), 0.99
Right foot	−1.23 (−1.70, −0.77), < 0.0001^a^	−1.34 (−2.12, −0.57), 0.0005^a^	0.11 (−0.72, 0.94), 0.94
Left foot	−1.33 (−1.85, −0.81), < 0.0001^a^	−1.28 (−2.15, −0.42), 0.003^a^	−0.04 (−0.97, 0.89), 0.99

*Note:* Values represent group contrasts (EMM differences with 95% confidence intervals) derived from an additive linear mixed‐effects model (LMEM) including fixed effects for participant group and applied pressure level (damage). As no group × damage interaction was included in the model, group contrasts are constant across pressure levels and are reported as effects adjusted for damage. *p*‐values are Tukey‐adjusted for multiple pairwise comparisons within each contrast family. Pain intensity was rated on a 0–10 numeric rating scale (NRS), and pinprick forces are expressed in millinewtons (mN). Control group sizes reflect 1:1 age‐ and sex‐matching to each PwP subgroup.

Abbreviations: EMM, estimated marginal mean; LMEM, linear mixed‐effects model; mN, millinewtons; NRS, numeric rating scale; PwP, people with Parkinson's disease.

^a^ Indicates a statistically significant difference (*p* < 0.05).

**TABLE 4:** Estimated marginal mean (EMM) differences in pressure pain thresholds (PPT) across participant groups and body regions.

Body region	PwP with pain (*n* = 27) vs. healthy controls (*n* = 27)	PwP without pain (*n* = 8) vs. healthy controls (*n* = 8)	PwP with pain (*n* = 27) vs. PwP without pain (*n* = 8)
Right neck	77.5 (16.3–138.8), 0.01^a^	165.3 (66.3, 264.2), 0.0007^a^	−87.7 (−193.2, 17.7), 0.12
Left neck	92.3 (24.1–160.4), 0.01^a^	180.0 (73.7, 286.4), 0.0006^a^	−87.8 (−198.6, 23.1), 0.14
Right arm	99.2 (42.7–155.8), 0.0004^a^	146.2 (58.4, 233.9), 0.0007^a^	−46.9 (−138.0, 44.1), 0.42
Left arm	79.0 (22.2–135.8), 0.005^a^	145.9 (57.8, 233.9), 0.0008^a^	−66.9 (−158.2, 24.4), 0.19
Right foot	90.8 (31.5–150.0), 0.002^a^	77.3 (−17.4, 172.0), 0.13	13.4 (−87.0, 114.0), 0.94
Left foot	82.1 (30.0–134.1), 0.001^a^	109.6 (29.9, 189.2), 0.01^a^	−27.5 (−109.3, 54.3), 0.69

*Note:* Values represent group contrasts (EMM differences with 95% confidence intervals) derived from a linear mixed‐effects model (LMEM) including participant group as the fixed effect. *p*‐values are Tukey‐adjusted for multiple pairwise comparisons within each contrast family. PPT values are expressed in kilopascals (kPa). Control group sizes reflect 1:1 age‐ and sex‐matching to each PwP subgroup.

Abbreviations: EMM, estimated marginal mean; kPa, kilopascals; LMEM, linear mixed‐effects model; PPT, pressure pain threshold; PwP, people with Parkinson's disease.

^a^ Indicates a statistically significant difference (*p* < 0.05).

### 3.1 Demographic Information of the Study Participants

Thirty‐five age‐ and sex‐matched individuals with PwP (mean age, 64.8 ± 10.3 years) and 35 healthy controls (mean age, 64.7 ± 10.3 years) were recruited during the study period. Among the PwP group, 27 participants (77.1%) reported experiencing pain or sensory disturbances lasting 3 months or more. For details on the recruitment process and participant characteristics, see Figure 1 and Table 1.

### 3.2 Demographic and Clinical Features of PwP and Healthy Controls

No significant difference was observed in MoCA scores between PwP and healthy controls (*p* = 0.08). The MoCA scores among PwP were evenly distributed across three levels (28, 29 and 30), whereas 60% of healthy controls scored 30. The MDI rating scale revealed higher MDI scores in PwP than in healthy controls (*p* < 0.01). However, no significant differences were observed in the MDI ICD‐10 Algorithm scores. While healthy controls reported no core or accompanying symptoms, PwP reported symptoms in both categories, with *p*‐values of 0.08 and 0.12, respectively. Clinical characteristics, including UPDRS scores, are presented in Table 1.

### 3.3 Dynamic Mechanical Allodynia (Brush Test)

#### 3.3.1 Both PwP Groups and Healthy Controls

Using Fisher's exact test, no significant differences in DMA were observed between PwP and healthy controls across the examined body regions (Table 2). *p*‐values of 0.05 were observed for the left arm and left leg, whereas no significant differences were observed for the remaining body regions. Table 2 summarizes the results and shows which participants scored ≥ 1, indicating the predefined threshold for DMA.

### 3.4 Static Mechanical Hyperalgesia (Pinprick Test)

#### 3.4.1 PwP With Pain and Healthy Controls

**FIGURE 2:**
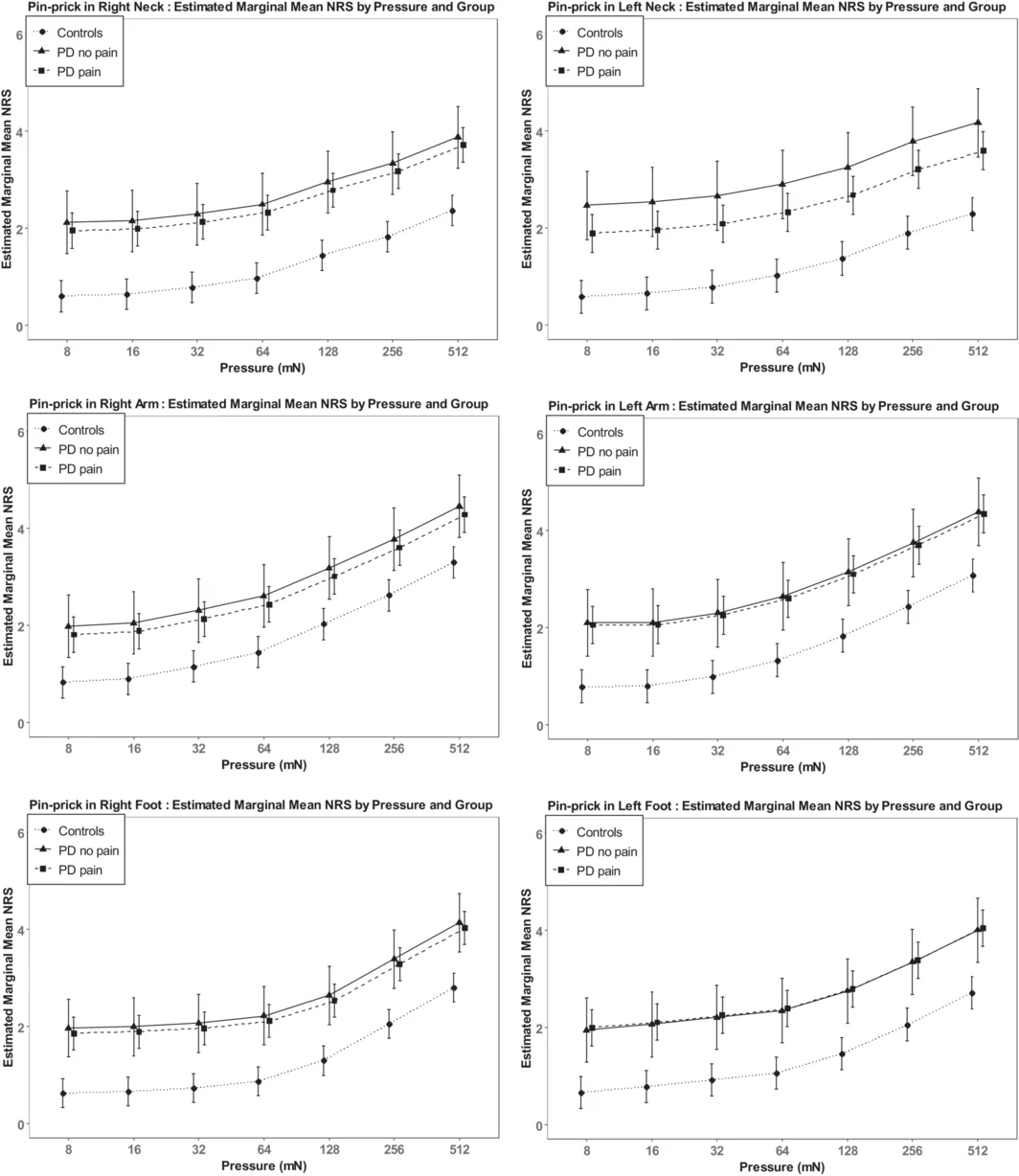
Pain ratings (NRS) in response to pinprick stimulation at increasing stimulus intensities in PwP with pain, PwP without pain and healthy controls across six body regions. Data are shown for the right neck, left neck, right arm, left arm, right foot and left foot. Healthy controls are indicated by dotted lines with circular markers, PwP without pain by solid lines with triangular markers, and PwP with pain by dashed lines with square markers. Lines represent estimated marginal means (EMM) with 95% confidence intervals derived from the fitted linear mixed‐effects models (LMEM) based on the observed data. Across all body regions, both PwP groups showed significantly higher pain ratings compared with healthy controls, whereas no significant differences were observed between PwP with and without pain. The *x*‐axis displays the applied pinprick forces (mN), and the *y*‐axis shows EMM pain intensity ratings on the numeric rating scale (NRS). The *x*‐axis is non‐linear, with the spacing between applied forces increasing twofold at each step. EMM, estimated marginal means; LMEM, linear mixed‐effects models; mN, millinewtons; NRS, numeric rating scale; PwP, people with Parkinson's disease.

As shown in Table 3 and Figure 2, LMEM analysis revealed significantly increased sensitivity to pinprick stimulation in PwP with pain compared with healthy controls across all body regions, consistent with the presence of static mechanical hyperalgesia. Significant differences were observed for the neck, arms and feet (all *p* < 0.001). Table 3 presents the EMM differences, 95% confidence intervals and *p*‐values.

#### 3.4.2 PwP Without Pain and Healthy Controls

Similarly, LMEM analysis showed significantly increased pinprick sensitivity in PwP without pain compared with healthy controls across all body regions, indicating the presence of hyperalgesia (Table 3, Figure 2). Significant differences were observed for the neck, arms and feet (all *p* < 0.05). Details of the EMM differences, 95% confidence intervals and *p*‐values are provided in Table 3.

#### 3.4.3 Comparison of PwP With and Without Pain

According to the LMEM analysis, no differences in pinprick sensitivity were observed between PwP with and without pain across any body region (Table 3, Figure 2). All comparisons were non‐significant, and the 95% confidence intervals crossed zero for all body regions. The EMM differences, 95% confidence intervals and *p*‐values are presented in Table 3.

#### 3.4.4 Comparison of Disease‐Onset Side vs. Contralateral Side in PwP With and Without Pain

Within‐subject comparisons based on LMEM analysis revealed no differences in pinprick sensitivity between the disease‐onset side and the contralateral side in either PwP group. No lateral differences reached statistical significance, and confidence intervals consistently crossed zero across all regions. Details of the estimated marginal mean (EMM) differences, 95% confidence intervals and *p*‐values are provided in Table S1.

### 3.5 Mechanical Pressure Hyperalgesia (Pressure Pain Threshold)

#### 3.5.1 PwP With Pain and Healthy Controls

**FIGURE 3:**
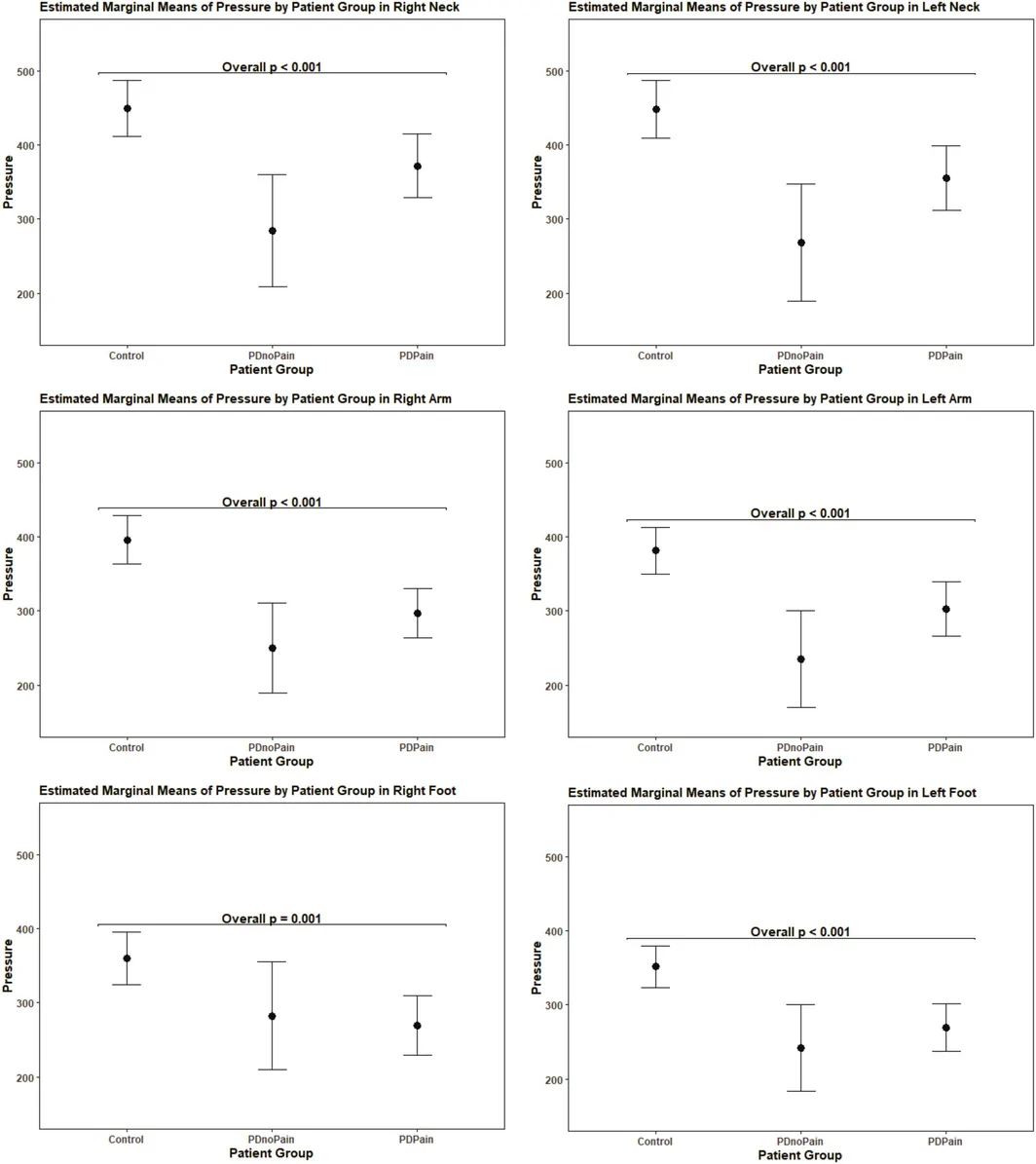
Pressure pain threshold (PPT) across participant groups and body regions. Estimated marginal means (EMMs) derived from linear mixed‐effects models (LMEMs) are shown for healthy controls, PwP without pain and PwP with pain across six body regions (right and left neck, arms and feet). Points represent EMMs, and error bars indicate 95% confidence intervals. Overall group effects derived from LMEMs are indicated above each panel. Pairwise comparisons were adjusted using Tukey's method. EMM, estimated marginal means; LMEM, linear mixed‐effects models; PDnoPain, Parkinson's disease without pain; PDPain, Parkinson's disease with pain; PPT, pressure pain threshold.

As shown in Figure 3, LMEM analysis revealed significantly lower PPT in PwP with pain than in healthy controls across all examined body regions, indicating heightened mechanical pressure sensitivity. Significant group differences were observed for the neck, arms and feet (all *p* ≤ 0.01). Table 4 presents the EMM differences, 95% confidence intervals and *p*‐values.

#### 3.5.2 PwP Without Pain and Healthy Controls

LMEM analysis further demonstrated significantly lower PPT in PwP without pain compared with healthy controls across most examined body regions, except for the right foot (Figure 3). Significant differences were observed for the neck, arms and left foot (all *p* ≤ 0.01). Detailed results, including the EMM differences, 95% confidence intervals and *p*‐values, are presented in Table 4.

#### 3.5.3 Comparison of PwP With and Without Pain

LMEM analysis revealed no significant differences in PPT between PwP with and without pain across any examined body region (Figure 3, Table 4). All comparisons were non‐significant, and the 95% confidence intervals crossed zero for all body regions. Further details of the LMEM analysis, including the EMM differences, 95% confidence intervals and *p*‐values, are provided in Table 4.

#### 3.5.4 Comparison of Disease‐Onset Side vs. Contralateral Side in PwP With and Without Pain

When comparing the disease‐onset and contralateral sides within subjects, LMEM analysis revealed no lateral differences in PPT in either PwP group. The absence of lateral asymmetry was supported by non‐significant comparisons and 95% confidence intervals consistently crossing zero. Detailed results, including the EMM differences, 95% confidence intervals and *p*‐values, are presented in Table S2.

## 4 Discussion

This study investigated somatosensory processes in PwP using selected QST measures. The main findings were increased pinprick sensitivity and generally lower PPT in both PwP with pain and without pain compared with individually age‐ and sex‐matched healthy controls. No differences in pinprick sensitivity or PPT were observed between PwP with and without pain or between the disease‐onset and contralateral sides. In addition, no differences in DMA were observed between PwP and healthy controls.

These findings indicate both expected and unexpected patterns, including mechanical hyperalgesia without corresponding allodynia and absence of lateralised sensory differences at this very early disease stage. Under normal conditions, brush stimulation is conveyed by low‐threshold Aβ‐fibres and perceived as touch, whereas pinprick stimulation and pressure algometer activate nociceptive Aδ‐ and C‐fibres and are perceived as pain (Mücke et al. 2021). The absence of group differences in DMA, together with the observed mechanical hyperalgesia, may therefore suggest early alterations in nociceptive processing, while Aβ‐fibre–mediated non‐nociceptive processing appears relatively preserved. Consistent with this interpretation, mechanical hyperalgesia was observed already at baseline, suggesting altered pain processing even among PwP without chronic pain. These early nociceptive abnormalities may reflect altered central pain modulation, potentially involving dysfunction in basal ganglia–thalamocortical circuitry or reduced dopaminergic modulation of nociceptive processing, both known to be affected early in PD (Tinazzi et al. 2010; Wasner and Deuschl 2012).

Although none of the participants met the ICD‐10 criteria for clinical depression, PwP exhibited significantly higher MDI scores than healthy controls. Previous studies have demonstrated an association between depressive symptoms and increased pain severity in PD (Mao et al. 2015; Valkovic et al. 2015). Accordingly, it cannot be excluded that even subclinical depressive symptoms contributed to altered pain processing and may have influenced the observed hyperalgesia. This potential confounding factor should therefore be considered when interpreting the findings.

Despite the increased pinprick sensitivity and lower PPT, only a small number of PwP (three to five patients per tested area) showed signs of DMA (NRS ≥ 1), while only one healthy control reached this threshold. The brush test revealed no significant differences between PwP and healthy controls across any body regions. Although *p*‐values of 0.05 were observed for the left arm and left leg, these did not meet the predefined threshold for statistical significance.

These findings contrast with a previous study in early‐stage PD (≤ 5 years) (Andersen et al. 2015), in which DMA was observed, and 58% of patients exhibited brush‐evoked discomfort, with significant differences between the arms and the lower back region. While our earlier observations suggested increased pain perception to non‐painful stimuli, this was not replicated in the present cohort of PwP. The discrepancy may reflect the very early disease stage in the current cohort and supports a progression model in which nociceptive abnormalities emerge early, whereas DMA develops later.

In line with Fründt et al. (2019), this study found no significant group‐level difference in DMA, although a small proportion of PwP showed signs of allodynia (NRS ≥ 1). Both studies examined similarly aged populations; however, most PwP in Fründt et al. were classified at Hoehn and Yahr Stage 2, whereas 51% of the present cohort were Stage 1. Methodological differences may also contribute to the divergent findings, including variations in brush protocols and the timing of assessment. As only a few studies have assessed DMA using brush strokes in PwP, direct comparisons remain limited. In addition, DMA may emerge later in the disease course than the nociceptive hypersensitivity detected by pinprick and PPT. Based on the present findings, there is no evidence that brush testing reliably detects DMA in PwP, suggesting limited utility as an indicator of altered sensory processing in early‐stage PD.

The pinprick test demonstrated mechanical hyperalgesia across all seven tested pinprick weights and all tested body regions in both PwP groups compared with healthy controls. Hyperalgesia was observed not only in PwP with pain, but also in those without daily pain, suggesting subclinical alterations in nociceptive processing already in the early, drug‐naïve stages of PD. These findings are consistent with our previous results in PwPD with a disease duration of ≤ 5 years (Andersen et al. 2015), where increased pain sensitivity to pinprick stimulation was also observed. While the present study demonstrated significant differences across all tested body regions, the previous study reported significant differences primarily in the lower back. Nevertheless, when all tested regions were analysed collectively, PwPD in the previous study rated pain intensity higher than healthy controls across all seven pinprick stimuli.

Several factors may explain the differences between the two studies. Participants in the current study were younger and exclusively drug‐naïve, whereas the previous cohort included patients with established PD. Additionally, the larger sample size in this study may have facilitated the detection of regional differences not observed previously. Overall, these findings support mechanical hyperalgesia as a consistent feature of PD‐related sensory abnormalities.

The present findings indicate that PwP exhibit mechanical hyperalgesia and that pain ratings increase with higher pinprick weights despite randomized stimulus presentation, supporting a coherent stimulus–response relationship.

In contrast to Fründt et al. (2019), who applied pinprick stimuli in a balanced order and evaluated mechanical pain sensitivity using DFNS‐based *z*‐scores relative to an external reference group, we directly compared pain ratings between PwP and individually age‐ and sex‐matched healthy controls. This different analytical approach, along with a larger sample size (35 vs. 19), may explain why significant group differences were detected in the present study but not in Fründt et al.'s study. Taken together, these findings suggest that pinprick testing may detect early mechanical hyperalgesia and reflect PD‐related nociceptive abnormalities in the drug‐naïve stage.

PwP also exhibited lower PPT than healthy controls across multiple body regions, indicating pressure hyperalgesia already in the drug‐naïve stage of PD. Significant group differences were observed in both arms as well as in the neck and feet, except for the right foot in PwP without pain. These findings extend our previous observations (Andersen et al. 2015), in which reduced PPT was detected in the dominant hand in PwPD with a disease duration of ≤ 5 years, whereas no differences were observed in the nondominant hand or lower back. In the present study, pressure hyperalgesia was observed across multiple body regions, which may reflect differences between the two studies, including sample size (35 vs. 12), patient characteristics and the number of regions tested.

In contrast, no significant group differences in PPT were observed (Fründt et al. 2019). Methodological differences may partly explain this, as PPT was assessed using a faster pressure increase rate (50 kPa/s) and analysed using DFNS‐based *z*‐scores relative to an external reference cohort, while controls were group‐matched rather than individually matched. Differences in stimulation parameters and analytical strategy may therefore have reduced sensitivity to detect group‐level differences compared with direct comparisons of absolute pain thresholds between individually matched patients and controls. Taken together, the presence of both pressure and pinprick hyperalgesia supports the presence of generalized mechanical nociceptive hypersensitivity already in the early, drug‐naïve stage of PD. Accordingly, PPT may represent a sensitive measure of pressure hyperalgesia in early PD and may complement pinprick testing in assessing nociceptive abnormalities. Correspondingly, Boura et al. reported increased spinal nociception in early PD, reflected by reduced nociceptive flexion reflex thresholds, and proposed that reduced descending mesencephalic dopaminergic inhibitory control may facilitate nociceptive processing (Boura et al. 2017). This mechanism may also be relevant to the mechanical hyperalgesia observed in the present drug‐naïve cohort and may support an interpretation of altered central nociceptive processing, although our QST measures cannot directly determine the underlying central or descending mechanisms. The present findings may complement the nociceptive dimension of the PD‐PCS by demonstrating mechanical nociceptive abnormalities in PwP (Mylius et al. 2021; Tinazzi et al. 2025). This provides insight into sensory alterations at a very early disease stage, before initiation of dopaminergic treatment.

## 5 Limitations

Several limitations should be considered. First, the comparison between PwP with and without pain was unequal (27 vs. 8 participants), which may have reduced the ability to detect subgroup differences despite clear differences between PwP and healthy controls. Recruiting newly diagnosed, drug‐naïve patients without pain was challenging, as pain and sensory disturbances were already prevalent at diagnosis. Consequently, the relatively small number of participants without pain may have limited the statistical power to detect differences between the two PwP groups. Accordingly, comparisons between the two PwP subgroups should be considered exploratory and interpreted with caution.

Second, a validated PD‐specific pain scale was not used to classify PwP with and without pain, which may limit the clinical interpretation of the subgroup analyses.

Third, no lateralised differences in sensory measures were detected, although PD is typically characterized by asymmetric motor symptom onset. Sensory asymmetries may emerge later in the disease course or require more pronounced motor asymmetry to become detectable. In very early, drug‐naïve stages, alterations in pain sensitivity may instead be bilateral, which may explain the absence of side‐specific differences in the present study.

Fourth, although the present sub‐study is embedded within The Aalborg PD Pain Project, a prospective longitudinal observational cohort study, the current analysis is based solely on cross‐sectional baseline data obtained before initiation of dopaminergic treatment. Consequently, no conclusions can be drawn regarding the temporal development of sensory abnormalities or whether subgroup differences become more pronounced over time. Longitudinal analyses of follow‐up data from the overarching project will be necessary to address these questions.

Fifth, although QST is a standardized method, pain ratings rely on subjective assessment and may be influenced by cognitive and affective factors, including attention, mood and task understanding. Future studies may benefit from combining QST with objective measures such as neuroimaging or autonomic markers.

Finally, the highly selected cohort of newly diagnosed PwP with preserved cognitive function and restricted comorbidity and medication use may limit the generalizability of the findings to the broader PD population, particularly to patients with more advanced disease or substantial comorbidity.

## 6 Conclusion

This study highlights early sensory dysfunction in PwP, with consistent evidence of mechanical hyperalgesia assessed by pinprick and PPT testing, even in individuals who do not report chronic pain. In contrast, no differences in DMA were observed, suggesting that nociceptive alterations (Aδ/C‐fibre–mediated processing) may precede abnormalities in non‐nociceptive (Aβ‐fibre–mediated) processing in early PD, possibly reflecting early alterations in central nociceptive processing. While asymmetry in pain perception was not observed at baseline, this may reflect early bilateral alterations in nociceptive processing, with lateralised differences potentially becoming more apparent with disease progression. Together, these findings support the use of nociceptive QST measures for characterizing altered sensory processing in early‐stage PwP. Future longitudinal studies will be essential to clarify the temporal relationship between sensory dysfunction, motor progression and the development of pain in PD, and to determine whether early sensory alterations predict later pain burden.

## Author Contributions

M.S.A., L.M.O., B.M. and P.G. conceived the study and designed the methodology. M.S.A. conducted the experiments and collected the data. M.S.A. and G.D.‐D. performed the data analysis. M.S.A. wrote the original draft. M.S.A., L.M.O., B.M., K.J. and P.G. supervised and critically revised the manuscript. All authors discussed the results and commented on the manuscript. All authors reviewed and approved the final version of the manuscript.

## Funding

This work was supported by the Danish Parkinson Association (Ph.D. salary) and by Helsefonden (grant number 15‐B‐0179) and Heinrich Kopps Legat (support for investigations and equipment). The funders had no role in the study design, data collection, analysis or interpretation of the data, preparation of the manuscript or decision to submit the manuscript for publication.

## Conflicts of Interest

The authors declare no conflicts of interest.

## Supporting information


**Table S1:** Estimated marginal mean (EMM) differences in static mechanical pain sensitivity (pinprick testing) between the disease‐onset side and the contralateral side across body regions in PwP. Values represent within‐subject differences (EMM differences with 95% confidence intervals) derived from linear mixed‐effects models (LMEMs). Disease‐onset side comparisons reflect within‐subject differences between the symptom onset side and the contralateral side in PwP. *p*‐values are Tukey‐adjusted for multiple comparisons. EMM, estimated marginal mean; LMEM, linear mixed‐effects model; PwP, people with Parkinson's disease.
**Table S2:** Estimated marginal mean (EMM) differences in pressure pain thresholds (PPT) between the disease‐onset side and the contralateral side across body regions in PwP. Values represent within‐subject differences (EMM differences with 95% confidence intervals) derived from linear mixed‐effects models (LMEMs). Disease‐onset side comparisons reflect within‐subject differences between the symptom onset side and the contralateral side in PwP. *p*‐values are Tukey‐adjusted for multiple comparisons. EMM, estimated marginal mean; LMEM, linear mixed‐effects model; PPT, pressure pain threshold; PwP, People with Parkinson's disease.
**Methods S1:** Supplementary statistical analysis.

## Data Availability

The data supporting the findings of this study are available from the corresponding author upon reasonable request.
