# Supplementary material for: Early Pain and Sensory Dysfunction in Drug‐Naïve Parkinson's Disease: Evidence From Quantitative Sensory Testing
Source: Eur J Pain. 2026 Sep 23;30(9):e70403. doi: 10.1002/ejp.70403 (PMC13598729; doi:10.1002/ejp.70403)
Supplement: Supplementary file 1 [file EJP-30-0-s001.docx]

## Supplementary Information

**MethodsS1**

**Supplementary Statistical Analysis**

All data were recorded and managed using REDCap electronic data capture tools (version 13.1.37) hosted at the North Denmark Region. Data validation rules, including range checks and required fields, were implemented to minimize entry errors, and manual data checks were performed to enhance data quality.

All statistical analyses and graphical visualizations were performed in R (version 4.3.3; R Foundation for Statistical Computing, Vienna, Austria) using the packages nlme for linear mixed-effects models (LMEMs), emmeans for estimated marginal means (EMMs), and ggplot2 for graphical visualization.

Baseline participant characteristics were compared between groups using Fisher’s exact test for categorical variables and unpaired t-tests for continuous variables. Continuous variables are presented as mean ± standard deviation (SD).

Given the limited number of pathological brush test scores (NRS ≥ 1), Fisher’s exact test was used to assess differences in brush-evoked pain between PwP and healthy controls, ensuring appropriate handling of sparse data.

LMEMs were used for the analyses of the pinprick and PPT data. Separate LMEMs were fitted for each anatomical location, defined by body part and side (left/right), using restricted maximum likelihood (REML).

For the pinprick analysis, the final LMEM included participant group and applied pressure level as fixed effects. Group-specific residual variances were modelled using a varIdent variance structure to allow for heteroscedasticity between participant groups.

For the PPT analysis, participant group was included as the sole fixed effect.

For both analyses, random intercepts were specified for matched pairs and participants nested within matched pairs. This random-effects structure accounted for the dependency introduced by the age- and sex-matched study design and for repeated observations within participants.

Model selection for the pinprick analysis was performed separately for each body part and side using maximum likelihood estimation. Specifically, a model including the interaction between participant group and applied pressure level was compared with a simpler additive model containing only the main effects. A likelihood ratio test showed that inclusion of the interaction term did not significantly improve model fit, and the additive model was therefore retained as the final model.

EMMs were derived from the final LMEMs using the emmeans package in R. For the pinprick analysis, pairwise contrasts between participant groups were estimated across applied pressure levels. For the PPT analysis, pairwise contrasts in PPT were estimated between participant groups.

To assess side-related differences within the PD groups, separate LMEMs were fitted in which side (disease onset side vs. contralateral side) replaced participant group as the fixed effect. For the pinprick analysis, a model including the interaction between side and applied pressure level was compared with an additive model using maximum likelihood estimation and a likelihood ratio test. As the interaction term did not significantly improve model fit, the additive model including side and applied pressure level as fixed effects was retained. Side was included as the sole fixed effect in the PPT analysis. The random-effects structure was identical to that used in the participant-group analyses.

P-values for pairwise comparisons were adjusted using Tukey’s method to control the familywise error rate.

Model diagnostics were performed to assess LMEM assumptions. Residual-versus-fitted plots, quantile–quantile (QQ) plots of residuals, and QQ-plots of random effects were examined. These diagnostics indicated no major violations of model assumptions.

**Table S1** Estimated marginal mean (EMM) differences in static mechanical pain sensitivity (pinprick testing) between the disease-onset side and the contralateral side across body regions in PwP. Values represent within-subject differences (EMM differences with 95% confidence intervals) derived from linear mixed-effects models (LMEMs). Disease-onset side comparisons reflect within-subject differences between the symptom onset side and the contralateral side in PwP. P-values are Tukey-adjusted for multiple comparisons. Abbreviations: EMM, estimated marginal mean; LMEM, linear mixed-effects model; PwP, People with Parkinson’s disease.

**Disease Onset Side vs. Contralateral Side (n = 35)**

-0.08 (-0.28, 0.11), 0.39

-0.08 (-0.28, 0.11), 0.39

0.18 (-0.05, 0.40), 0.12

0.18 (-0.05, 0.40), 0.12

0.04 (-0.24, 0.32), 0.77

0.04 (-0.24, 0.32), 0.77

**Table S2** Estimated marginal mean (EMM) differences in pressure pain thresholds (PPT) between the disease-onset side and the contralateral side across body regions in PwP. Values represent within-subject differences (EMM differences with 95% confidence intervals) derived from linear mixed-effects models (LMEMs). Disease-onset side comparisons reflect within-subject differences between the symptom onset side and the contralateral side in PwP. P-values are Tukey-adjusted for multiple comparisons. Abbreviations: EMM, estimated marginal mean; LMEM, linear mixed-effects model; PPT, pressure pain threshold; PwP, People with Parkinson’s disease.

**Disease Onset Side vs. Contralateral Side (n = 35)**

7.42 (-10.6, 25.5), 0.41

7.42 (-10.6, 25.5), 0.41

-12.6 (-34.9, 9.7), 0.26

-12.6 (-34.9, 9.7), 0.26

4 (-11.5, 19.5), 0.60

4 (-11.5, 19.5), 0.60
